# Avian vampire fly (*Philornis downsi*) mortality differs across Darwin’s finch host species

**DOI:** 10.1038/s41598-021-94996-7

**Published:** 2021-08-04

**Authors:** Lauren K. Common, Petra Sumasgutner, Rachael Y. Dudaniec, Diane Colombelli-Négrel, Sonia Kleindorfer

**Affiliations:** 1 College of Science and Engineering, Flinders University, Bedford Park, SA 5001 Australia; 2 Department of Behavioral and Cognitive Biology, Konrad Lorenz Research Center, Core Facility for Behavior and Cognition, University of Vienna, Vienna, Austria; 3 Department of Biological Sciences, Macquarie University, Sydney, NSW 2109 Australia

**Keywords:** Ecology, Environmental sciences

## Abstract

In invasive parasites, generalism is considered advantageous during the initial phase of introduction. Thereafter, fitness costs to parasites, such as host-specific mortality, can drive parasites towards specialism to avoid costly hosts. It is important to determine changes in host specificity of invasive populations to understand host-parasite dynamics and their effects on vulnerable host populations. We examined changes in mortality in the introduced avian vampire fly (*Philornis downsi*) (Diptera: Muscidae), a generalist myasis-causing ectoparasite, between 2004 and 2020 on Floreana Island (Galápagos). Mortality was measured as the proportion of immature larvae found upon host nest termination. Over the time period, the avian vampire fly was most abundant and had low mortality in nests of the critically endangered medium tree finch (*Camarhynchus pauper*) and had the highest mortality in nests of hybrid tree finches (*Camarhynchus* spp.). Low larval mortality was also found in small tree (*Camarhynchus parvulus)* and small ground finch (*Geospiza fuliginosa)* nests. Selection could favour avian vampire flies that select medium tree finch nests and/or avoid hybrid nests. Overall, the finding of differences in avian vampire fly survival across host species is parsimonious with the idea that the introduced fly may be evolving towards host specialisation.

## Introduction

Niche breadth is a fundamental concept that underpins key hypotheses in species ecology^1–3^. The breadth of a niche is the set of conditions in which a species can persist, and can include dimensions such as habitat diversity and climatic variation^4^. In parasitic organisms, niche breadth is often synonymous with host specificity—i.e., the number of host species a parasite can infect, and parasites range from highly host specific to generalist^5–7^. Host specificity is mediated by host-parasite co-evolutionary processes^8^. Hosts and parasites enter an arms race in which they adapt and counter-adapt reciprocally at the expense of the other^9^. Hosts are selected to evade or resist the parasite, whereas parasites evolve to more efficiently exploit their host^6^. Higher virulence (damage to the host) and host specificity can lead to increased exploitation of the host by the parasite^10^. Selection for greater host exploitation may break down when parasite fitness is reduced, whereby high exploitation of hosts leads to premature host mortality, leading to decreased parasite growth and fecundity, or increased parasite mortality^11,12^.

Host specificity presents trade-offs for the parasite. Generalist parasites tend to occur on host species that are phylogenetically closely related^7,13–15^. Nonetheless, they incur the cost of maintaining variation in life history, genetic and behavioural traits that enable exploitation of different host species^16^. This relationship can be further complicated in host hybrid zones, where hybrids can be more or less resilient to parasite populations^17^. Despite high host encounter rates due to wide host ranges, generalist parasite populations exhibit slower geographic expansion rates compared to specialist populations^16^. The occurrence of parasite generalism or specialism is influenced by the costs and benefits inherent to occupying different host ranges, including mortality rates in parasite populations associated with particular host species^18^. For blood feeding parasites, for example, the costs of generalist feeding can push species towards specialisation because of the variation in host blood properties and nutritional value for the parasite^19^. High mortality risk or a lack of nutritional value in specific hosts can drive parasites to specialise on hosts that optimise their fitness^20^. Generalist parasites may have a selective advantage when colonising novel environments given their capacity to switch hosts if a primary host population declines, which can increase their chance of persistence despite a range of establishment challenges^21,22^.

When a generalist parasite colonises a novel environment and suite of potential host species, the differences in fitness due to altered selection creates a window of opportunity to study niche and host specialisation shifts under changing evolutionary pressures. While selection may initially favour a generalist strategy to maximise initial spread upon colonisation, specialisation is favoured in parasites that are capable of host choice^23,24^. In fact, generalism is rare compared to specialism, with most species parasitising only one or a few host species^20,25,26^. Compared with generalists, specialist parasites have been shown to evolve more quickly in response to evolving host defences and have fewer deleterious alleles present in their gene pool^9,27^. Fitness differences and resulting mortality of parasites in certain host species can drive parasites to specialise on ideal hosts that minimise energetic and fitness costs. When a given optimal host species is abundant, predictable^28^, accessible^26^, and energetically efficient for the parasite^24^, host specialisation is expected to evolve rapidly^23^. Host specialisation can be favoured when fitness trade-offs are present and in the presence of multiple viable hosts that vary in fitness costs for the parasite^23,29^.

Here, we consider the case of the avian vampire fly (*Philornis downsi*) (Diptera: Muscidae) (Dodge and Aitken 1968), a generalist myiasis-causing invasive parasitic fly of the Galápagos Islands. First observed in a Darwin’s finch nest in 1997 on Santa Cruz Island, the avian vampire fly is currently known to parasitise nestlings in all 18 studied bird species on the Galápagos Islands^30,31^. Low host defences due to immunological naivety to this type of parasite^32,33^ and close taxonomic clustering of Darwin’s finches^7^ may have facilitated its rapid spread across hosts and the archipelago^31,34^. Avian vampire fly larvae consume the blood and tissue of developing birds in the nest^35^, causing high in-nest mortality in their hosts^31,36^. Nestlings that survive the parasitism often have permanently deformed nares, affecting their song and foraging strategy^37,38^ (Kleindorfer et al. unpublished). Given the apparent ubiquity of the avian vampire fly across Galápagos passerine species and the increasing number of avian vampire fly larvae and pupae per host nest in studies carried out between 2000 to 2013^36,39^, theory predicts that parasite generalism should prevail if there are negligible resource differences (i.e. nutritional value or fitness costs to parasites) between host species. However, host specialisation or host preference should occur if there are differences in fitness costs for the avian vampire fly between host species.

Nestling mortality in Darwin’s finches caused by blood-sucking avian vampire fly larvae can be high (55% on average), with hosts dying younger in recent years^36,39^. Yet, some Darwin’s finch species appear better able than others to tolerate the impacts of avian vampire fly parasitism^36,40–42^, perhaps because of differences in brood size, such as between ground (*Geospiza*) and tree (*Camarhynchus*) finches^43^. Smaller broods have higher parasite loads per nestling and hence suffer higher nestling mortality^44,45^. On Santa Cruz Island, nestling mortality caused by avian vampire fly larvae has shifted across the past decade in warbler finch (*Certhidea olivaceae*) and small tree finch (*Camarhynchus parvulus*)^46,47^. Initially, during 2000–2005, warbler finches had on average more larvae per nest than small tree finches (41 ± 6 compared to 23 ± 3)^45,48^, with the pattern reversing during 2010–2014^46^. During 2012–2014, 56% of small tree finch nestlings died due to vampire fly parasitism, 71% of nests lost the whole brood before nestlings reached 7 days old, compared to 37% mortality in warbler finch nestlings^46^. The differences in host mortality and intensity (total number of parasites present in the nest) between warbler finches compared with small tree finches on Santa Cruz Island might be due to changes in the oviposition behaviour by the vampire fly or the behaviour of the host, which remains to be further explored (but see^49^).

Host tolerance of parasitism is also dependent on environmental conditions. For example, droughts and heavy rainfall may exacerbate the negative impact of avian vampire fly parasitism when hosts are unable to compensate with increased nestling feeding rates or experience elevated numbers of parasites in nests^46,50^. Periods of high rainfall, such as El Niño years, have been associated with increased numbers of avian vampire fly larvae in nests across host species^40,45,51^. High rainfall years have also been associated with increased hybrid recruitment in *Camarhynchus* tree finches on Floreana Island^52^. Current hybridisation patterns occur as female medium tree finches (*C. pauper*) pair with male small tree finches (*C. parvulus*), resulting in sex‐specific gene flow and the existence of a hybrid swarm^53^. Hybrid nests have significantly fewer avian vampire fly larvae with up to 60% fewer parasites per nest than their parental species^42^. Parents of nests that had the greatest genetic admixture (therefore, greater hybrid assignment probability) had the fewest avian vampire fly larvae. However, the mechanisms driving these intensity differences are not yet known^42^.

Here, we explore changes in avian vampire fly larval mortality across time and host species. We suggest that if there are changes in avian vampire fly larval mortality in different host species, this could indicate selection on the avian vampire fly to diverge and specialise, or to avoid particular hosts. Our long-term data offer a unique opportunity to describe early co-evolutionary processes in a generalist parasite across a suite of hosts in its invasive range, which has implications for the evolution of host specificity. We analyse data spanning non-consecutive 17-years of avian vampire fly specimens collected from Floreana Island, Galápagos, to examine changes in in-nest mortality and survival when parasitising three host species and a hybrid cluster: small ground finch (*Geospiza fuliginosa*), small tree finch (*C. parvulus*), medium tree finch (*C. pauper*), and the hybrid tree finch (*C. pauper* × *C. parvulus*, including hybrids that have backcrossed to one of the parent species^53^). In a comparison of samples collected from host nests between 2004 and 2020, we predict that (1) avian vampire fly intensity (i.e. total number per nest) has increased over time regardless of the host species based on previous research^39^; (2) avian vampire fly larval mortality has increased since 2004—we predict a positive relationship between vampire fly larval mortality and year, and mortality and nestling age at death (the age at which the last nestling dies), as early host death (i.e. early termination of resources)^39^ results in younger parasites upon nest termination; (3) avian vampire fly larval mortality will increase with increasing annual rainfall, as heavy rainfall decreases host survival^46,50^ and, (4) avian vampire fly larval mortality differs between host species due to (a) differences in brood size between small ground finch and the tree finch species^43^ and (b) differences in parasite-induced nestling mortality between the small and medium tree finches and the hybrid tree finch given differences in parasite intensity between these host species^42^.

## Methods

### Study system

This study was conducted on Floreana Island, Galápagos Archipelago, and followed long-term field protocols as described below^39^. The field work was conducted during the Darwin’s finch breeding season in the highlands (01° 17′ S, 090° 27′ W) between the months of January and April in ten non-consecutive seasons spanning 17 years: 2004, 2005, 2006, 2008, 2010, 2012, 2013, 2014, 2016 and 2020. We collected avian vampire fly specimens from the nests of the small ground finch, small tree finch, medium tree finch, and the hybrid *Camarhynchus* tree finch^53–55^. Host species were first determined morphologically, and hybrid tree finches were retrospectively confirmed via genetic analyses^53,54^. Due to this approach, data for hybrid *Camarhynchus* finches are only available for the years 2006, 2010, 2012, 2013 and 2014. Floreana rainfall data (sum of annual rainfall; mm) were collected via satellite sourced from CPC Global Unified Precipitation Data provided by NOAA/OAR/ESRL PSD, Boulder, Colorado, USA, downloaded from the Galápagos Vital Signs website by the Galápagos Conservancy^56^.

### Study species

The avian vampire fly is an obligate myiasis-causing parasite of birds that feeds on the blood and tissue of developing nestlings^44^. Non-parasitic adult flies feed upon decaying vegetable matter, ovipositing their eggs in active bird nests^57–59^. Upon hatching, first and early second instar larvae move to the naris and ear canals of nestlings to feed on blood and keratin^35^. Late second and third instar larvae feed externally on nestlings at night, residing in the base of the nest during the day^35,60,61^. Reports of development times of larval instars vary between field and lab reared specimens, with pupation occurring after 4–10 days of feeding^39,62^. Upon host fledging or death, third instar larvae pupate in the base of the nest, forming frothy cocoons and emerging as adults within 7–18 days^39,62^.

### Nest monitoring and vampire fly collection

We analysed data from 280 Darwin’s finch nests on Floreana Island with all avian vampire fly specimens per nest collected and stored in ethanol following well-established field protocols^39^. Small tree finch (n = 64), hybrid tree finch (n = 34), medium tree finch (n = 55) and small ground finch (n = 127) nests were monitored for activity and brood size every 3 days during incubation and every 2 days during the nestling phase. Males of these host species build new display nests at which they sing for each new nesting event^63^. The female either selects a display nest or selects a male and they build a new display nest together^63^. Incubation lasts ~ 14 days and, if successful, nestlings fledge the nest approximately 12–14 days after hatching. Brood size was determined using a borescope to view inside the nest once nestlings had hatched. After nesting activity had finished (i.e., nest termination, either through death of the nestling or fledging), the nest was collected and dismantled within 24 h to count the number of avian vampire fly offspring within the nest. Nestling age at death was known for a subset of nests (n = 105) from hatching date or visual aging of the nestlings via borescope. In all sampling years (except for four nests in 2004 and 2005), nestlings found dead in the nest were soaked in 70% ethanol for 24 h to allow first instar larvae within the nestling nares or ear canals to float and be collected. We generally only collect ~ 8 1st and 2nd instar using this method from ~ 8% of nestlings soaked. The 1st instar larvae reside inside the nares for the first two days post-hatch and nestlings tended to survive until d7 post-hatch during 2004 and 2005^39^. All avian vampire fly larvae, pupae, puparia and adult flies were stored in 70% ethanol within 24 h of collection.

Larval specimens of 241 nests were assigned an age class via observation using a dissecting microscope, following instar identification protocols^35,49^. Parasite intensity was calculated as the total number of larvae, pupae, puparia and adult flies within a nest. Mortality in the avian vampire fly larvae was measured as the proportion of immature (first and second instar) larvae in the nest at the time of host resource termination^64^. This measure accounts for the possibility of third instars and pupae fully developing into adult flies following host termination^65^. This measure also provides an estimate of parasite mortality per host nest, given that first and second larval instars are unable to continue development in the absence of nutrition^65,66^.

### Statistical analysis

All models were fitted using R version 4.0.3^67^ with the packages lme4^68^, MASS^69^, and car^70^, and were visualised with lattice^71^, ggplot2^72^ and effects^73^. Total number of avian vampire fly offspring per nest (log transformed to fulfil the assumption of normality) was analysed in relation to study year, annual rainfall and the Darwin’s finch species and the interaction between year and rainfall as fixed effects with a linear regression model on the full data set (n = 280).

For the corresponding analyses considering different age classes of the parasite, we had a smaller dataset of n = 241. We repeated the analysis of total intensity in relation to year and rainfall on this subset of data to confirm the same pattern across both data sets. We analysed the total number of first and second instar larvae, third instar larvae, total larvae, pupae and puparia in relation to year and annual rainfall similar to the total number of vampire flies*,* but as count data with Generalized Linear Models (GLMs) with negative binomial distribution and log link function to correct for overdispersion. Throughout we tested for linear and quadratic relationships of year and rainfall and their additive and interactive effects on the total avian vampire fly intensity*,* first and second instar larvae, third instar larvae, total larvae, pupae and puparia. Based on the principles of parsimony (the largest amount of variance explained with the minimum number of predictors^74^), we then selected the model structure that best described our measures of parasite loads at different developmental stages.

Avian vampire fly larval mortality was modelled using the column bind (‘cbind’) function specifically designed to fit proportion data in logistic regression models with the number of larvae in the first and second larval instar as binomial denominator, and a quasibinomial distribution and a logit link function to correct for overdispersion. We fitted the key response variable avian vampire fly larval mortality to two different data sets: (1) considering all Darwin’s finch nests on Floreana Island for which we identified larvae to age class (n = 241); and (2) considering nests where nestling age at death was known (n = 106), with nestling age at death as an additional co-variate. This second analysis accounts for changes in parasite intensities within nests according to nestling age at death^44^; and highlights interspecific host differences in vampire fly larval mortality even when accounting for host age at death^39^. We fitted the study year, annual rainfall and the Darwin’s finch species and the interactions (year × rainfall) as fixed effects. Initially, we also controlled for brood size, but this additional predictor did not reveal any significant result and was dropped from the final model to improve sample size (from n = 191 to n = 241 in the data set without brood size). We removed non-significant interaction terms from the models to simplify the statistical approach and interpretation of the results and to ensure a valid interpretation of the remaining additive effects. The effect of host genus (*Geospiza* and *Camarhynchus*), excluding hybrid tree finches to remove the effect of hybridisation (n = 213), was analysed using the full model of mortality in relation to year and annual rainfall and their interaction as fixed effects.

All quantitative variables were scaled (standardized to mean = 0 and standard deviation = 1) to bring the variables to comparable dimensions and to facilitate the correct interpretation of effect sizes for interaction terms^75^. Residual distributions of the models were inspected visually to assess model fit (diagnostic plots produced by the ‘plot’ function in the ‘base’ package: residuals versus fitted values and normal Q–Q plot displaying the theoretical quantiles versus standard Pearson residuals). Throughout, we report model effect sizes (estimates ± SE, derived from the summary function); presented χ2 and *p*-values are based on an ANOVA Table of Deviance using Type III Wald χ2 tests (ANOVA function in ‘car’ package). No random factors were considered, as there were no repeated measurements in the data. We tested for correlations of fixed effects beforehand but did not find any indication for co-linearity in our data.

### Permits

Permission to conduct this study was given by the Galápagos National Park and Charles Darwin Research Center, permit no. MAE-DNB-CM-2016-0043, and Flinders University, permit no. E480/19.

## Results

### Avian vampire fly intensity

**Figure 1:**
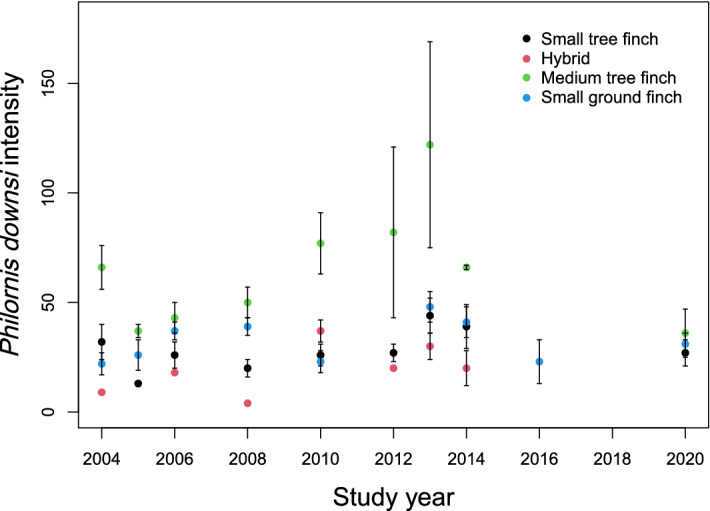
Number (mean ± SE) of avian vampire flies (*Philornis downsi*) per nest of Darwin’s finch species per year on Floreana Island. Each Darwin’s finch species is denoted by a different colour.

We found no effect of year on avian vampire fly intensity (i.e., total number of parasites per nest) across the entire study period (LM, F_1, 275_ = 0.040, estimate 0.012 ± 0.02, *p* = 0.583, Table 1, Fig. 1). There was an effect of species: medium tree finches had the highest intensity of avian vampire flies per nest (57.1 ± 5.4 vampire flies per nest compared to *C. parvulus*: 29.9 ± 2.3; *Camarhynchus* hybrids: 26.6 ± 3.3; *G. fuliginosa*: 36.2 ± 2.0) (LM, F_3, 275_ = 3.038, estimate 0.254 ± 0.07, *p* < 0.001, Table 1; raw data in supplementary Table S2).

**Table 1:** Linear model for avian vampire fly (*Philornis downsi*) intensity in relation to year and host species collected between 2004 and 2020 from Darwin’s finch nests on Floreana.

*Philornis downsi* intensity (n = 280)	Estimate	SE	t value	Sum Sq	df	*P*-value	Sign
Intercept	*1.401*	*0.045*	*30.857*			< *0.001*	***
Year	0.012	0.022	0.549	0.040	1	0.583	
Hybrid^a^	− 0.086	0.078	− 1.115	3.038	3	< 0.001	***
Medium tree finch	0.254	0.067	3.796				
Small ground finch	0.041	0.056	0.728				

‘Rain’ did not feature into the most parsimonious model. Avian vampire flies collected from Darwin’s finch nests over 10 years across a 17-year period on Floreana Island.

^a^Species ‘small tree finch’ was used as a reference category. Note the response variable *Philornis downsi* intensity was log transformed to achieve normality and all quantitative input variables were scaled and centred. Intercept presented in italics. Sign = significance levels: ‘***’ < 0.001.

### Age class distribution and abundance

Analysis of avian vampire fly age classes revealed a significant quadratic relationship with year and the number of first and second instar larvae (GLM, ‘year’ term estimate: − 0.51 ± 0.14, *p* = 0.002, Table 2b, Figure S1), with numbers of first and second instar larvae peaking in approximately 2013. The number of first and second instar larvae was consistently higher in hybrid tree finches (Table S1). Furthermore, across all species, third instar larvae and the total number of larvae per nest increased until 2013 and decreased thereafter (GLM, third instar: ‘year’ term estimate: − 0.32 ± 0.10, *p* = 0.010; total larvae: ‘year’ term estimate: − 0.38 ± 0.10, *p* = 0.001, Table 2c,d, Figure S1). Conversely, the number of pupae and puparia per nest showed the opposite pattern, decreasing until 2013 and 2014 onwards (GLM, ‘year’ term estimate: 0.46 ± 0.10, *p* < 0.001; ‘year’ term estimate: 0.78 ± 0.24, *p* = 0.002, respectively, Table 2e,f, Figure S1). There was no significant effect of rainfall on the total intensity in these nests (LM, ‘rainfall’ term estimate: − 0.04 ± 0.03, *p* = 0.110, Table 2a, Figure S1) or on any other age class considered (i.e., rainfall did not feature in any other parsimonious model, neither in the linear nor quadratic relationship).

**Table 2:** Linear Models exploring the effects of rain and year on avian vampire fly (*Philornis downsi*) intensity and age class.

	Estimate	SE	t-value	Sum Sq	Df	*P*-value	Sign
**(a) *Philornis downsi* intensity (n = 241; log transformed)**							
(Intercept)	*1.42*	*0.03*	*54.73*			< *0.001*	***
Rain	− 0.04	0.03	− 1.60	0.42	1	0.110	

	Estimate	SE	z-value	LR χ^2^	Df	*P*-value	
**(b) *Philornis downsi* first and second larval instar (n = 241)**							
(Intercept)	*1.72*	*0.14*	*12.43*				
Year (linear)	− 0.06	0.14	− 0.45	12.69	2	0.002	**
Year (quadratic)	− 0.51	0.14	− 3.60				
**(c) *Philornis downsi* third larval instar (n = 241)**							
(Intercept)	*2.47*	*0.10*	*23.76*				
Year (linear)	0.01	0.10	0.12	9.13	2	0.010	*
Year (quadratic)	− 0.32	0.10	− 3.10				
**(d) *Philornis downsi* total larvae (n = 241)**							
(Intercept)	*2.86*	*0.10*	*28.49*				
Year (linear)	0.00	0.10	− 0.03	13.90	2	0.001	***
Year (quadratic)	− 0.38	0.10	− 3.79				
**(e) *Philornis downsi* pupae (n = 241)**							
(Intercept)	*2.45*	*0.10*	*24.98*				
Year (linear)	0.01	0.10	0.07	20.22	2	< 0.001	***
Year (quadratic)	0.46	0.10	4.71				
**(f) *Philornis downsi* puparia (n = 241)**							
(Intercept)	*0.80*	*0.24*	*3.39*				
Year (linear)	− 0.52	0.24	− 2.22	12.40	2	0.002	**
Year (quadratic)	0.78	0.24	3.31				

(a) response variable *P. downsi* infection intensity, log transformed to achieve normality; and Generalized Linear Models (negative binomial distribution) of (b) first and second larval instar; (c) third larval instar; (d) total number of larvae; (e) total number of pupae; and (f) total number of puparia for the different life stages of *P. downsi* in relation to year and rainfall (fitted in a linear or quadratic relationship). We show the most parsimonious model after considering linear and quadratic relationships of year and rainfall and their interaction. Analysis of Deviance Table (Type III tests). Avian vampire flies collected from Darwin’s finch nests over 10 years across a 17-year period (2004–2020) on Floreana Island.

Note all quantitative input variables were scaled and centred. A full intercept is only displayed for Linear Models and cannot be derived with the ANOVA function for Generalized linear models. Intercept presented in italics. Sign = significance levels: ‘***’ < 0.001; ‘**’ < 0.01; ‘*’ < 0.05; ‘.’ < 0.1.

### Larval mortality

**Figure 2:**
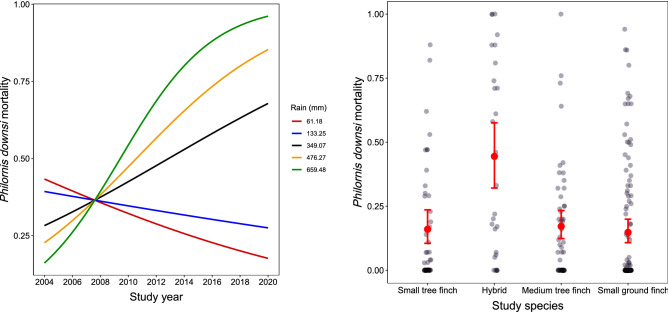
The relationship between avian vampire fly (*Philornis downsi*) in-nest larval mortality and (**a**) the interaction between study year and annual rainfall (sum in mm); and (**b**) the different Darwin’s finch species. Note the interaction is plotted for min (rainfall = 61.18 mm, red line), 1st quantile (rainfall = 133.25 mm), median (rainfall = 349.07 mm), 3rd quantile (rainfall = 476.27 mm) and max (rainfall = 659.48 mm, black dashed line) values; while the additive effect is plotted as effect sizes plus 95% CIs. Model details provided in Table 3.

**Figure 3:**
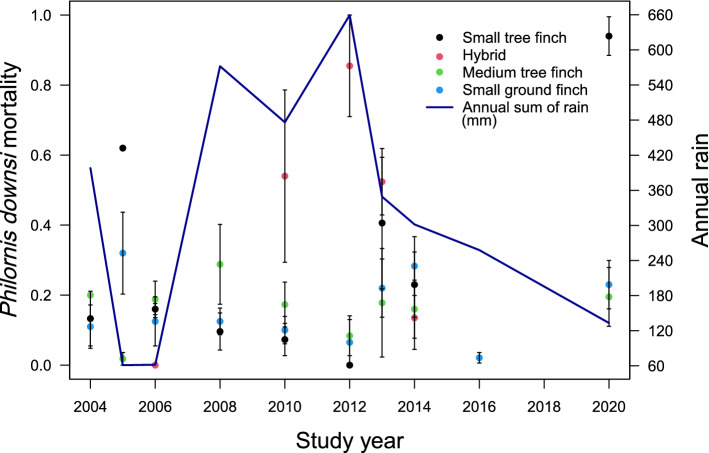
The relationship between avian vampire fly (*Philornis downsi*) in-nest mortality and year across the Darwin’s finch host species, with cumulative annual rainfall on Floreana Island. Dots represent the proportion of vampire fly larvae that died upon termination of the host and are labelled according to the four different Darwin’s finch species.

For all samples combined, there was an increase in the proportion of avian vampire fly larval mortality over time (‘year’ term estimate: 0.48 ± 0.19, *p* = 0.012, Table 3a) and during years with higher annual rainfall (‘rainfall’ term estimate: 0.40 ± 0.13, *p* = 0.002, Table 3a). However, these additive effects should be interpreted with caution due to their involvement in a significant interaction term (estimate 0.58 ± 0.18, *p* = 0.002; Fig. 2a). Earlier in the study period (2004–2008), when the years were drier (e.g., ~ 360 mm), vampire fly mortality was lower; while later in the study period (2010–2020), when the years were wetter (e.g., 400–650 mm), avian vampire fly mortality was higher (Fig. 1a, Fig. 3). Larval mortality did not differ between the small tree finch, medium tree finch or small ground finch, but was significantly higher in hybrid nests (least square means and post-hoc contrasts Table 3b,c, Fig. 2b). Larval mortality did not differ between *Camarhynchus* and *Geospiza* host nests when excluding hybrid tree finches (estimate ‘genus’ term − 0.15 ± 0.24, *p* = 0.532; Table 4).

**Table 3:** Generalized linear model for (a) avian vampire fly (*Philornis downsi*) in-nest mortality in relation to year and rainfall (interaction term) and species; (b) lsmeans (least squares means; extracted with the ‘emmeans’ package) and (c) post-hoc contrasts for vampire fly in-nest mortality between Darwin’s finch species.

(a) *Philornis downsi* in-nest mortality (n = 241)	Estimate	SE	t-value	LR χ^2^	df	*P*-value	Sign
Intercept	− *1.659*	*0.245*	− *6.758*			< *0.001*	***
Year	0.477	0.188	2.533	6.660	1	0.010	*
Rain	0.402	0.128	3.128	9.997	1	0.002	**
Hybrid^a^	1.436	0.366	3.924	23.483	3	< 0.001	***
Medium tree finch	0.084	0.316	0.267				
Small ground finch	− 0.093	0.304	− 0.305				
Year × Rainfall	0.577	0.182	3.170	10.496	1	0.001	**

(b) lsmeans between species	Probability	SE	LCL	UCL			
Small tree finch	0.16	0.03	0.11	0.24			
Hybrid	0.45	0.07	0.32	0.58			
Medium tree finch	0.17	0.03	0.12	0.23			
Small ground finch	0.15	0.02	0.11	0.20			

(c) Post-hoc contrast	Odds ratio	SE	z-ratio	*P*-value	Sign		
Small tree finch/Hybrid	0.24	0.09	− 3.92	0.001	**		
Small tree finch/Medium tree finch	0.92	0.29	− 0.27	0.993			
Small tree finch/Small ground finch	1.10	0.33	0.31	0.990			
Hybrid/Medium tree finch	3.87	1.27	4.12	0.000	***		
Hybrid/Small ground finch	4.62	1.54	4.59	< .0001	***		
Medium tree finch/Small ground finch	1.19	0.33	0.65	0.915			

Avian vampire flies collected from Darwin’s finch nests in 10 sampling years across a 17-year period (2004–2020) on Floreana Island.

^a^Species ‘small tree finch’ was used as a reference category. × indicates an interaction term

Dispersion Parameter for quasibinomial family taken to be 12.381. Intercept presented in italics. Sign = significance levels: ‘***' < 0.001; ‘**' < 0.01; ‘*' <0.05; lsmeans intervals are back-transformed from the logit scale and post-hoc contracts were performed on the log-odds ratio scale following the tukey method.

**Table 4:** Generalized linear model for avian vampire fly (*Philornis downsi*) in-nest mortality in relation to year and rainfall (interaction term); and genus (*Camarhynchus* sp. n = 108; *Geospiza* sp. n = 115), excluding *Camarhynchus* hybrids.

*Philornis downsi* in-nest mortality (n = 213)	Estimate	SE	t-value	LR χ^2^	df	*P*-value	Sign
Intercept	− *1.605*	*0.151*	− *10.614*			< *0.001*	***
Year	0.462	0.192	2.405	6.007	1	0.014	*
Rain	0.377	0.130	2.894	8.467	1	0.004	**
Ground finch (*Geospiza fuliginosa*)	− 0.150	0.240	− 0.625	0.392	1	0.531	
Year × Rain	0.562	0.184	3.059	9.763	1	0.002	**

Avian vampire flies collected from Darwin’s finch nests in 10 sampling years over a 17-year period (2004–2020) on Floreana Island.

^a^Genus *Camarhynchus* sp. ‘tree finch’ was used as a reference category. × indicates an interaction term.

Note all quantitative input variables were scaled and centred. Dispersion Parameter for quasibinomial family taken to be 12.421. Intercept presented in italics. Sign = significance levels: ‘***' < 0.001; ‘**' < 0.01; ‘*' < 0.05.

When analysing nests where the nestling age at death was known, we found a strong effect of nestling age at death on larval mortality. Larval mortality increased as nestling age at death decreased (estimate − 0.34 ± 0.15, *p* = 0.025, Table 5, Figure S2c). The interaction effect of year and rainfall on mortality was marginally non-significant (estimate 0.38 ± 0.20, *p* = 0.056, Table 5, Figure S2a). Avian vampire fly mortality was significantly higher in hybrid hosts (estimate 1.36 ± 0.41, t = 3.30, *p* < 0.001, Table 5, Figure S2b). Larval mortality did not differ between the small ground, small tree, or medium tree finch (Figure S2b).

**Table 5:** Generalized linear model for avian vampire fly (*Philornis downsi*) in-nest mortality in relation to year and rainfall (interaction term) and species, including the co-variate ‘nestling age at death, ranging from 1 to 14 days).

*Philornis downsi* in-nest mortality	Estimate	SE	t-value	LR χ^2^	df	*P*-value	Sign
*Intercept*	− 1.128	0.283	− 3.985			< *0.001*	***
Year	0.116	0.228	0.508	0.390	1	0.609	
Rain	0.194	0.168	1.152	0.915	1	0.248	
Hybrid^a^	1.361	0.413	3.296				
Medium tree finch	− 0.189	0.383	− 0.494	21.040	3	< 0.001	***
Small ground finch	− 0.063	0.369	− 0.170				
Nestling age at death	− 0.344	0.152	− 2.270	5.500	1	0.018	*
Year × Rainfall	0.378	0.201	1.877	4.112	1	0.056	

Including this co-variate reduces our data set to n = 106. Avian vampire flies collected from Darwin’s finch nests in 10 sampling years over a 17-year period (2000–2020) on Floreana Island. Significant estimates indicated in bold.

^a^Species ‘small tree finch’ was used as a reference category. × indicates an interaction term.

Note all quantitative input variables were scaled and centred. Dispersion Parameter for quasibinomial family taken to be 12.273. Intercept presented in italics. Sign = significance levels: ‘***' < 0.001; ‘*' < 0.05.

## Discussion

In this study, we tested patterns of larval mortality in avian vampire fly, a generalist myiasis-causing parasite of Darwin’s finches, across time and host species. We did not find a significant increase in parasite mortality across time, but there were clear differences in parasite mortality across host species. Parasite mortality was lowest in nests of the medium tree finch, and highest in hybrid finch nests, even when accounting for chick age at death^39^. If host-specific selection pressures on larval mortality continue or increase, the avian vampire fly may be selected to oviposit in optimal host nests, which may result in host specialisation.

Our results provide some support for the idea that *Camarhynchus* hybridisation may be an adaptive host response to thwart a novel parasite, in line with previous findings^42^. The Red Queen hypothesis is a powerful theoretical framework to predict host-parasite coevolutionary dynamics, and one expects that host-impacting change caused by the parasite is countered by the host, and vice versa^9^. The newly evolving Darwin’s finch and avian vampire fly system is consistent with the idea of oscillating evolutionary dynamics in the wild but requires additional research into genetic and behavioural mechanisms to more fully understand these patterns. Previous research has shown that: (1) during the first part of the decade from 2004 to 2013^39^, the average number of avian vampire flies per host nest increased and then stabilised; (2) one host species, the medium tree finch, consistently has the most avian vampire flies in the nest compared with other host species^40^; (3) the proportion of hybrid birds increased from 12% in 1998 to between 27 and 55% in later years, and hybrid hosts have the fewest avian vampire flies compared with other host species^52^; and here we show that (4) avian vampire fly mortality was highest in hybrid finch nests and lowest in the nests of the other host species (small ground, small tree and medium tree finches), even when accounting for nestling age at death. From the perspective of the parasite, it should avoid hybrid finch nests. The mechanisms that may drive host-seeking versus host-avoidance behaviours by the parasite are unknown. However, this study uncovers two concurrent scenarios whereby both parasite intensity and parasite mortality across hosts differed, especially between medium tree finch and hybrid tree finch nests during the early co-evolutionary stages of a host-parasite interaction.

In parasites that use multiple host species for different life stages, host generalism is the optimal strategy^76^. The avian vampire fly lives its parasitic life stages in a single host environment, and in this case, specialist offspring are predicted to be optimal to maximise arithmetic mean fitness^76^. The observation that different Darwin’s finch host species have different average numbers of avian vampire flies per nest, even immediately after host hatching, is in line with the idea of differentiated oviposition in certain hosts^42,46^. Despite specialisation, some specialist lineages hedge their bets by ovipositing in suboptimal hosts^76,77^. In the case of the avian vampire fly, genetic evidence has shown oviposition by multiple females in one host nest; also, females frequently lay fewer eggs than they are able to oviposit at a time, which the supports the idea of bet hedging by ovipositing in multiple nests^78^. However, it is currently unknown if females oviposit preferentially in specific host nests or whether there could be host-specific lineages of avian vampire fly.

Given that high rainfall is associated with more avian vampire flies in host nests^40,45,51^, we would expect to see an increase in competition between larvae during high rainfall years as more parasites compete for the same amount of resources^79,80^. Increased competition may lead to increased parasite mortality. However, in this study, we found lowest parasite mortality in nests of the host species with the most parasites, the critically endangered medium tree finch. The extreme fluctuations in rainfall within parasite lifetimes and across generations on the Galápagos Islands may favour environmental generalists that maintain optimal fitness levels with rainfall fluctuation^76^. Selection pressures from introduced pathogens can lead to swamping of local environmental adaptation in favour of immune response loci^81^. Therefore, there may be a trade-off between achieving optimal parasite fitness across multiple host species and the parasite’s capacity to tolerate environmental variation. In the avian vampire fly, such relationships are yet to be explored.

Host specialisation may ease the burden of parasitism in some host species yet may heighten the threat for other neighbouring species, particularly in host-limited, geographically restricted habitats, as occurring on Floreana Island^82^. Smaller, endangered populations, such as the medium tree finch, are more likely to have low genetic diversity with a reduced capacity to evolve in response to parasites^83^. The threat posed by the parasite is further exacerbated by the high intensity of avian vampire fly larvae found in medium tree finch nests. In comparison, the hybrid tree finch that is the result of recombination between the small and the medium tree finch may have increased genetic variation, which may offer novel genes on which selection can act to evolve resistance to parasitism^84,85^. Given the observation that female medium tree finch frequently pair with male small or hybrid tree finches rather than medium tree finch, and the potential for increased hybrid resilience^42,53^, the medium tree finch population may continue to decline, eventually resulting in only a hybrid swarm^17^. Hybrid recruitment, as measured by the proportion of yearling birds in the population, has remained stable across years since 2005, whereas medium tree finch recruitment rates declined across the same period, suggesting hybrid nestlings and/or fledglings may have a selective advantage over medium tree finch offspring^52^. Understanding host-specific parasite fitness in this system highlights the need for directed conservation efforts to more exploited hosts or those less likely to evolve parasite resistance mechanisms. Our results suggest that such mechanisms may be evolving in the *Camarhynchus* hybrid group, but at a cost to the medium tree finch population.

The effects of host hybridisation on both host and parasite fitness have mainly been documented in plant-parasite systems, as hybridisation is common in plant species^86^. These effects vary between systems. Host hybridisation can, for example, increase susceptibility to parasites, resulting in increased numbers of parasites and decreased hybrid fitness^17,87,88^. In other cases, host hybridisation increases host resistance and tolerance, decreasing parasite loads and increasing host fitness^17,89^. We see this latter pattern in the Darwin’s finch system, where hybrid tree finches tend to have fewer parasites per nest than their parent species^42^. In this study, using a sub-sample of nests for which we have accurate data on parasite age class, we also found a pattern of fewer parasites per nest in hybrid nests, though the difference in number of parasites across host species was not statistically significant. There is not much available data on parasite fitness in hybrid versus non-hybrid hosts, which is a research gap that requires attention. In a study on fungal pathogens infecting hybrid plant hosts, pathogens had a fitness advantage in hybrid hosts that was contingent on pathogen hybridisation^90^. In addition, the role of host hybrid fitness is expected to affect parasite fitness^91^. If host resistance and tolerance to parasites increases as the consequence of hybridisation, then parasite fitness could be higher in hybrid hosts able to sustain the parasite, or conversely, parasite fitness could be lower in hybrid hosts that deter parasites from ovipositing. More research is needed to explore different host-parasite evolutionary pathways under conditions of genetic introgression in host and/or parasite.

We don’t know why avian vampire fly larval mortality differed across hosts species in this study, but it is known that blood properties of host species can vary in nutritional gain for the parasite^19,28^. Mortality in second and third instar avian vampire fly larvae reared on chicken blood did not differ between formulated diets of varying nutrition, however development time to pupation was fastest on the diet with the highest nutritional value^62^. Decreased developmental time is advantageous when resources can be terminated quickly, such as when Darwin’s finch nestlings die young^39^, allowing more larvae to reach pupation faster and hence survive to adulthood in nutritionally optimal hosts. High mortality was found in first instar larvae reared on artificial diets and the possible contamination of the blood with pathogenic bacteria such as *Serratia* may be driving this high mortality^62^. S*erratia*, a genus with pathogenic species that affects myiasis-causing and muscid flies, was found to be uniquely associated with avian vampire flies parasitising warbler finches in a microbiome analysis of the fly^92^. Warbler finches in recent years had fewer avian vampire flies and lower host mortality compared to tree finches^46^. Research has further shown that the avian vampire fly microbiome differs significantly across Darwin’s finch host species, which is suspected to be associated with differences in finch diets within and across habitats^55,92–95^. Overall, the findings of this and previous research suggest that larval mortality may be driven by multiple factors, including host nutritional quality, habitat, and microbiome.

We found high parasite mortality in hybrid avian hosts, which we document in a generalist and recently introduced parasite to the Galapagos archipelago. The parasite did best in nests of the Floreana Island endemic, the medium tree finch. Theory predicts that the vampire fly should be selected to oviposit preferentially in medium tree finch nests, given that it has the highest pupation success in medium tree finch nests, and avoid hybrid finch nests where most of its offspring fail to pupate. Understanding the mechanisms by which the avian vampire fly avoids or selects host nests, invests in generalist or specialist offspring, or alters its strategy to survive in prevailing environmental conditions are at the forefront of research into this rapidly evolving host-parasite interaction system on the Galápagos Islands. Our study provides evidence for differential fitness of an invasive parasite in nests of different host species.

## Supplementary Information

Supplementary Information.

## Data Availability

All data analysed within this paper are available through the Dryad Digital Repository: 10.5061/dryad.9ghx3ffhw.
