# Supplementary material for: Avian vampire fly (Philornis downsi) mortality differs across Darwin’s finch host species
Source: Sci Rep. 2021 Aug 4;11:15832. doi: 10.1038/s41598-021-94996-7 (PMC8338931; doi:10.1038/s41598-021-94996-7)
Supplement: Supplementary file 1 — Supplementary Information. [file 41598_2021_94996_MOESM1_ESM.docx]

**Supplementary Material**

**Avian vampire fly (*Philornis downsi*) mortality differs across Darwin’s finch host species**

Lauren K. Common, Petra Sumasgutner, Rachael Y. Dudaniec, Diane Colombelli-Négrel, Sonia Kleindorfer

**Table S1:** Mean number of first and second instar larvae and total number of avian vampire flies (*Philornis downsi*) collected from nests of four different Darwin’s finch species across 17 years in the highlands of Floreana Island. N = the number of nests. Data are shown as mean ± SE.

|  | **Small tree finch** | | | **Hybrid tree finch** | | | **Medium tree finch** | | | **Small ground finch** | | |
| --- | --- | --- | --- | --- | --- | --- | --- | --- | --- | --- | --- | --- |
| Year | N | 1^st^/2^nd^ Instar | Total | N | 1^st^/2^nd^ Instar | Total | N | 1^st^/2^nd^ Instar | Total | N | 1^st^/2^nd^ Instar | Total |
| 2004 | 4 | 5.00 ± 2.89 | 44.25 ± 14.06 | 0 |  |  | 1 | 15.00 | 76.00 | 9 | 0.78 ± 0.78 | 17.56 ± 7.34 |
| 2005 | 1 | 8.00 | 13.00 | 0 |  |  | 5 | 0.80 ± 0.80 | 36.00 ± 4.90 | 8 | 9.88 ± 3.73 | 28.50 ± 5.14 |
| 2006 | 9 | 0.33 ± 0.33 | 36.56 ± 7.44 | 1 | 0.00 | 9.00 | 10 | 6.60 ± 1.89 | 44.10 ± 7.48 | 6 | 0.33 ± 0.33 | 43.58 ± 10.91 |
| 2008 | 9 | 2.00 ± 1.15 | 21.11 ± 3.62 | 0 |  |  | 10 | 6.10 ± 3.04 | 40.40 ± 10.76 | 25 | 4.56 ± 1.49 | 44.78 ± 5.78 |
| 2010 | 7 | 3.00 ± 2.21 | 27.86 ± 5.06 | 3 | 22.33 ± 7.69 | 46.33 ± 9.26 | 12 | 12.00 ± 6.45 | 40.58 ± 11.33 | 19 | 2.47 ± 1.47 | 18.11 ± 3.49 |
| 2012 | 2 | 0.00 ± 0.00 | 20.50 ± 6.50 | 2 | 22.00 ± 2.00 | 27.00 ± 7.00 | 5 | 7.60 ± 4.82 | 51.20 ± 28.51 | 0 |  |  |
| 2013 | 5 | 18.80 ± 10.56 | 43.80 ± 7.58 | 14 | 16.43 ± 4.57 | 30.07 ± 6.13 | 4 | 16.25 ± 14.95 | 47.50 ± 19.73 | 8 | 11.88 ± 5.37 | 45.75 ± 5.66 |
| 2014 | 7 | 7.29 ± 2.74 | 37.71 ± 10.38 | 8 | 5.50 ± 4.80 | 17.38 ± 5.95 | 3 | 11.33 ± 6.57 | 64.00 ± 7.21 | 8 | 9.25 ± 2.23 | 43.88 ± 8.16 |
| 2016 | 0 |  |  | 0 |  |  | 0 |  |  | 3 | 2.00 ± 2.00 | 28.00 ± 11.85 |
| 2020 | 8 | 4.00 ± 4.00 | 36.13 ± 8.81 | 0 |  |  | 4 | 1.00 ± 0.71 | 43.00 ± 11.01 | 21 | 1.57 ± 0.88 | 37.24 ± 5.46 |

**Table S2:** Mean *P. downsi* intensity, host nestling age at death and percentage of avian vampire fly (*Philornis downsi*) larval mortality from four Darwin’s finch host species between 2004 and 2020 in the highlands of Floreana Island. N = number of nests. Data are shown as mean ± SE.

| **Host** | **N** | ***P. downsi* intensity** | **Age at death** | **% *P. downsi* mortality overall** |
| --- | --- | --- | --- | --- |
| Small tree finch | 53 | 33.02 ± 2.89 | 6.46 ± 0.39 (N = 26) | 13.6 ± 3.1 |
| Hybrid tree finch | 28 | 27.21 ± 3.94 | 5.89 ± 0.51  (N = 19) | 41.9 ± 7.2 |
| Medium tree finch | 55 | 44.41 ± 4.52 | 6.64 ± 0.52  (N = 22) | 16.5 ± 3.0 |
| Small ground finch | 107 | 34.51 ± 2.40 | 6.52 ± 0.43  (N = 38) | 16.2 ± 2.4 |


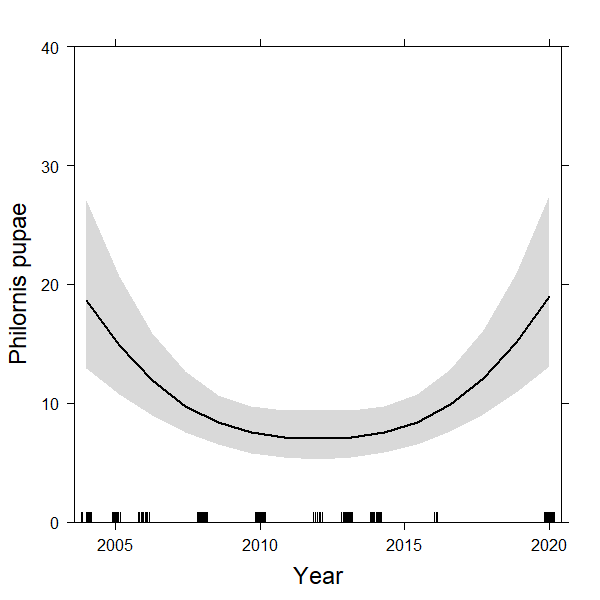

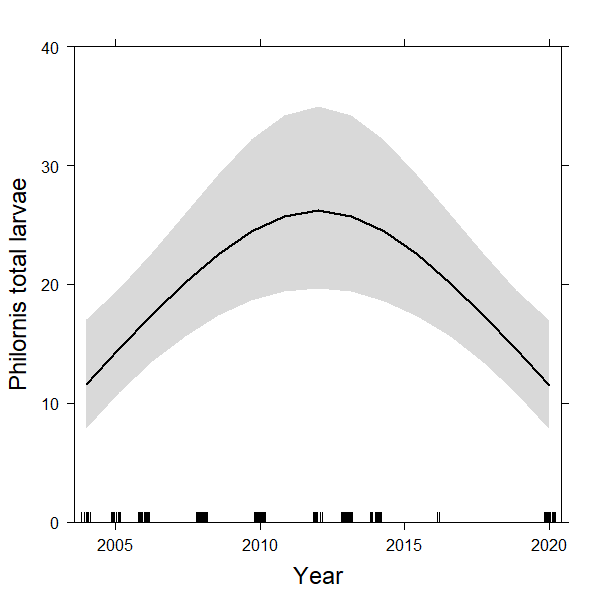

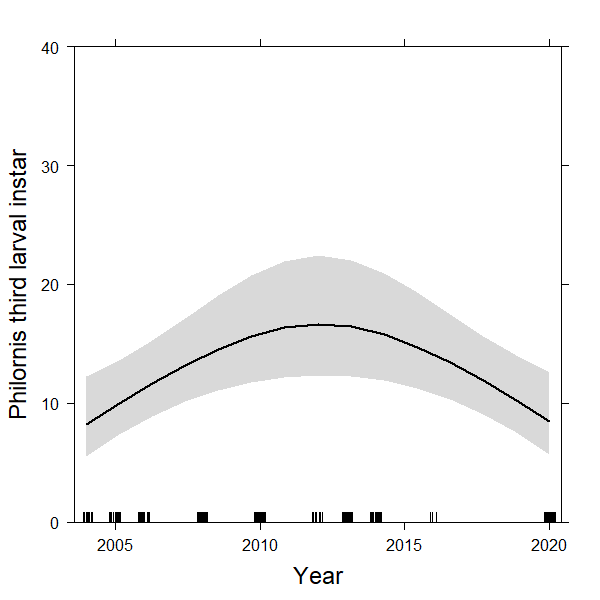

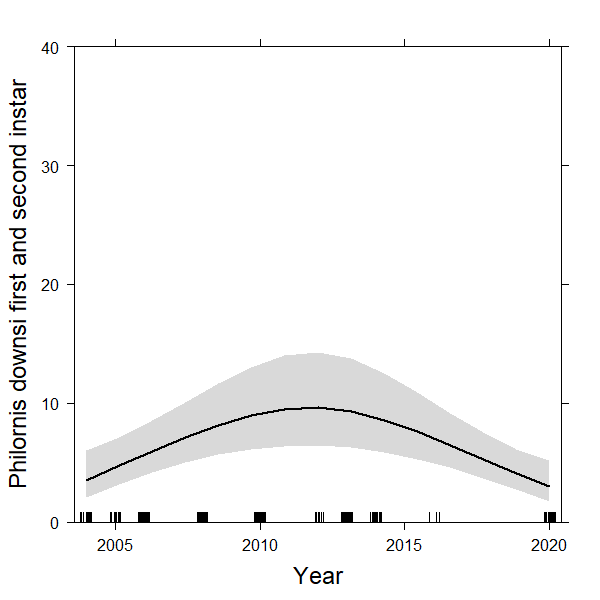

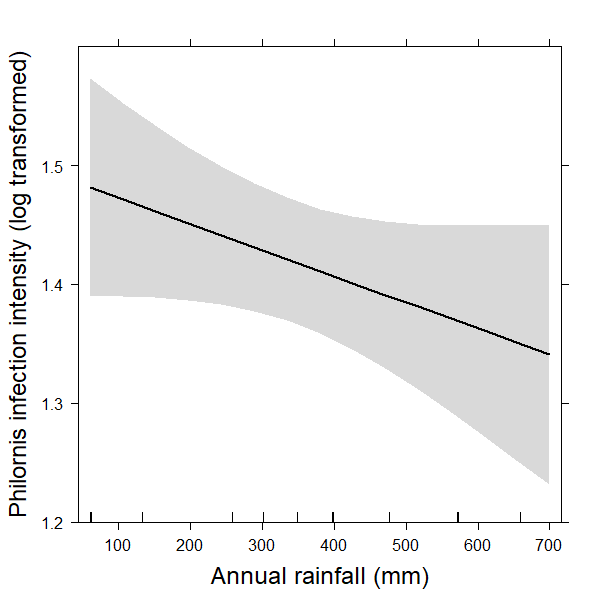

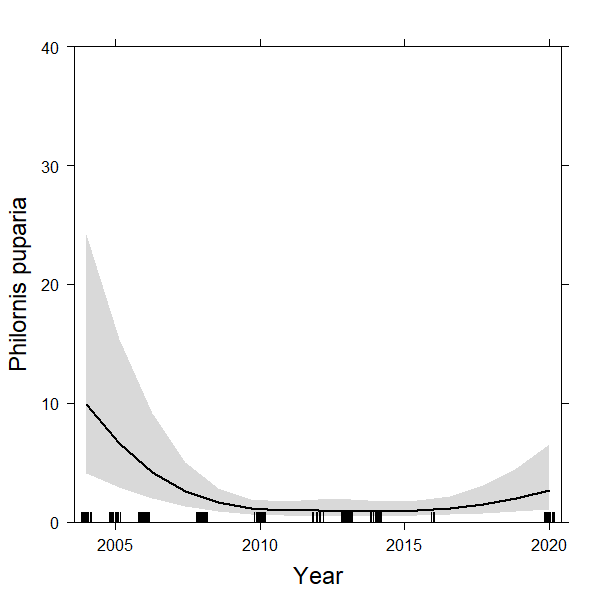


**Figure S1:** Relationship between avian vampire fly (*Philornis downsi*) infection intensity, first and second larval instar, third larval instar, total number of larvae and total number of pupae, year and annual rainfall from Darwin’s finch nests between 2004 and 2020 on Floreana Island (n = 241). Effect sizes plus 95% CIs; black bars on the x-axis represent sample size; model details provided in Table 2.


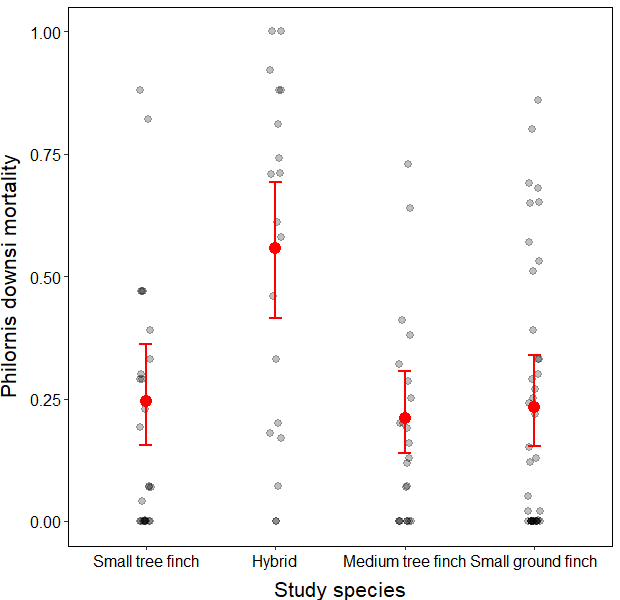

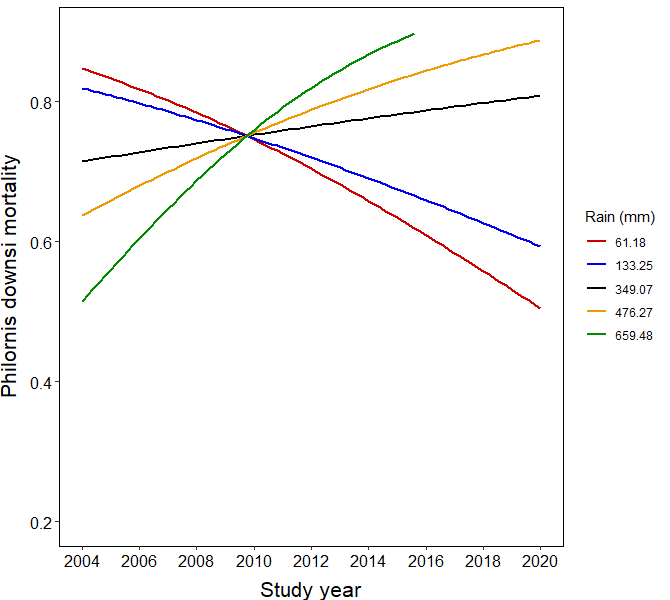


(b)

(a)


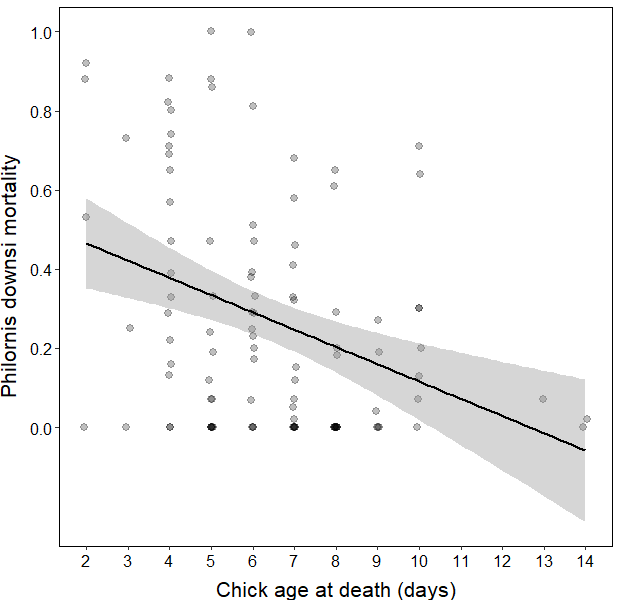


(c)

**Figure S2:** The relationship between avian vampire fly (*Philornis downsi*) in-nest larval mortality (n = 106) and (a) the interaction between study year and annual rainfall (sum in mm); (b) the different Darwin’s finch species; and (c) chick age at death (reducing the sample size to n = 106). Note the interaction is plotted for min (rainfall = 61.18 mm, red line), 1^st^ quantile (rainfall = 133.25 mm), median (rainfall = 349.07 mm), 3^rd^ quantile (rainfall = 476.27 mm) and max (rainfall = 659.48 mm, black dashed line) values; while the additive effect is plotted as effect sizes plus 95% CIs. Model details provided in Table 5.
